# Supplementary material for: The virulence regulator VirB from Shigella flexneri uses a CTP-dependent switch mechanism to activate gene expression
Source: Nat Commun. 2024 Jan 5;15:318. doi: 10.1038/s41467-023-44509-z (PMC10770331; doi:10.1038/s41467-023-44509-z)
Supplement: Supplementary file 1 — Supplementary Information [file 41467_2023_44509_MOESM1_ESM.pdf]

Supplementary information

The virulence regulator VirB from *Shigella flexneri* uses a CTP-dependent switch mechanism to activate gene expression

Sara Jakob, Wieland Steinchen, Juri Hanßmann, Julia Rosum, Katja Langenfeld, Manuel Osorio-Valeriano, Niklas Steube, Pietro I. Giammarinaro, Georg K. A. Hochberg, Timo Glatter, Gert Bange, Andreas Diepold, Martin Thanbichler

Supplementary figures

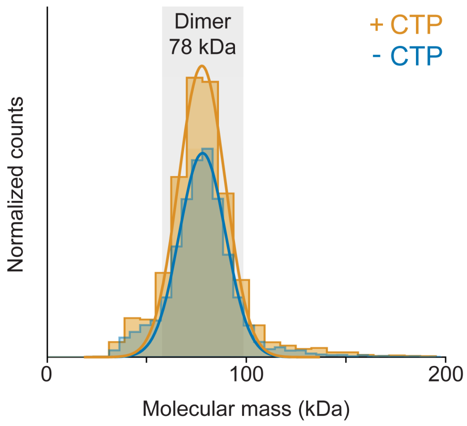

Supplementary Figure 1. Mass photometry analysis of the oligomeric state of VirB. The graph shows histograms of the measured molecular mass distributions of VirB particles in the absence or presence of CTP (1 mM) as well as the fitted normal distributions. The peaks obtained correspond to molecular masses of 78 ± 11.7 kDa (+ CTP) and 78 ± 11.6 kDa (- CTP).

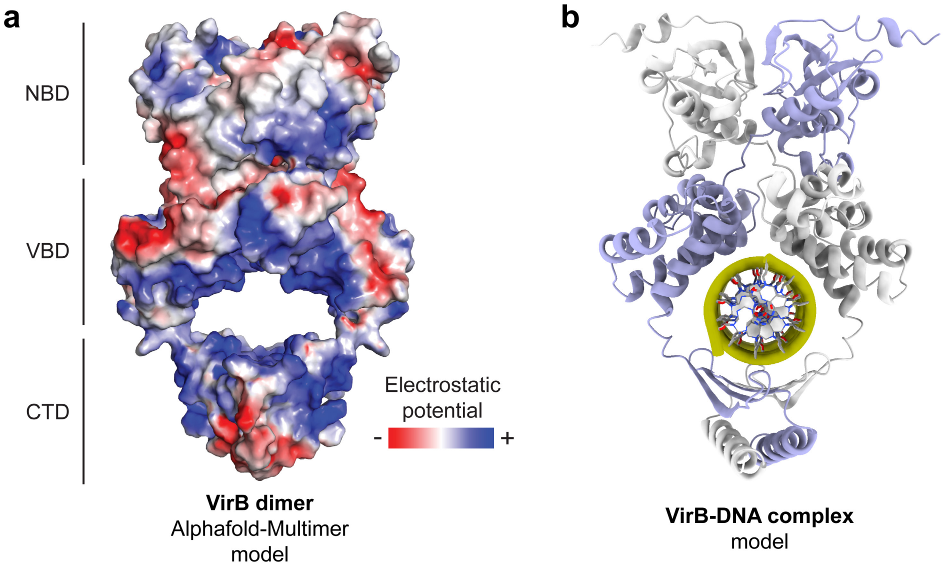

Supplementary Figure 2. Properties of the predicted non-specific DNA-binding region of VirB. (a) Electrostatic potential surface of VirB, determined with PyMOL (Schrödinger LLC, USA) and plotted onto a structural model of the VirB dimer, generated with AlphaFold-Multimer [1]. The nucleotide-binding domain (NBD), the *virS*-binding domain (VBD) and the C-terminal dimerization domain (CTD) are indicated. The color code is given on the right. **(b)** Model of the complex of a closed VirB dimer with non-specific DNA.

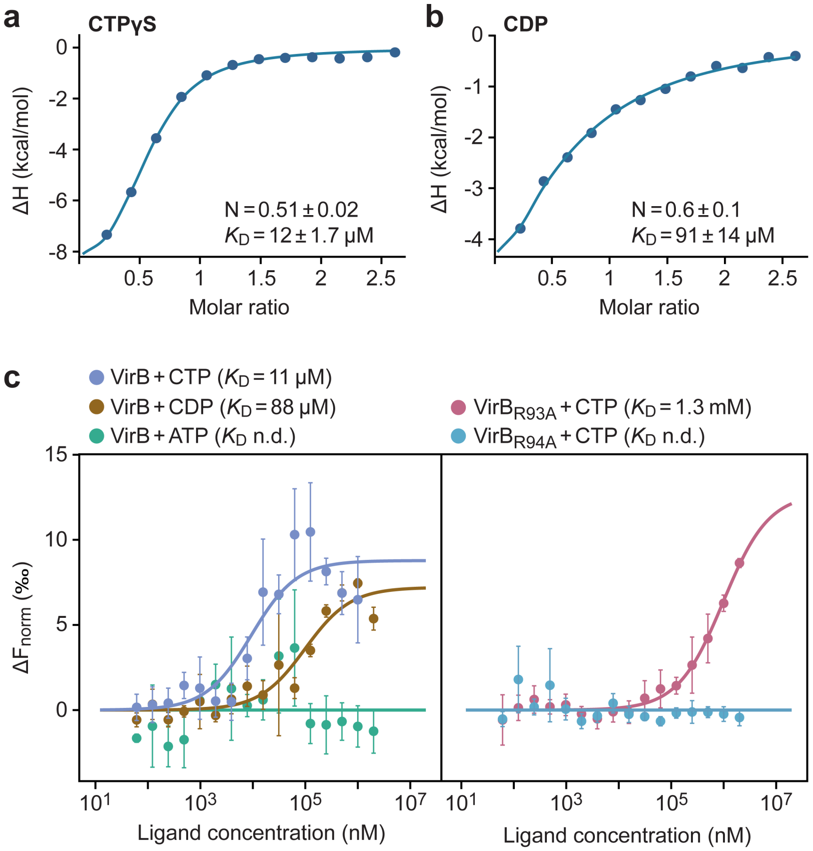

Supplementary Figure 3. Nucleotide-binding behavior of VirB. (a,b) Isothermal titration calorimetry analysis of the interaction of VirB with (a) CTPγS and (b) CDP. The graphs display the incremental enthalpy changes in the titrations shown in **Figure 2a,b**, corrected for the heats of dilution. Data were fitted to a one-set-of-sites binding model. **(c)** Microscale thermophoresis analysis of the nucleotide-binding behavior of VirB and mutant variants. Wild-type VirB, VirB-R93A and VirB-R94A were fluorescently labeled and then analyzed for their thermophoretic mobility in the presence of increasing concentrations of nucleotides. The data points represent the mean of the normalized ΔF values (± SD) obtained in n=3 (CTP binding to VirB-WT and VirB-R93A) or n=2 (all other reactions) independent experiments, each of which was performed in triplicate. To plot the binding curves, the results of all replicates were combined and fitted using MO Affinity Analysis v2.3 (Nanotemper, Germany). The K_D values were calculated as described in **Figure 2c**. Source data are provided as a Source Data file.

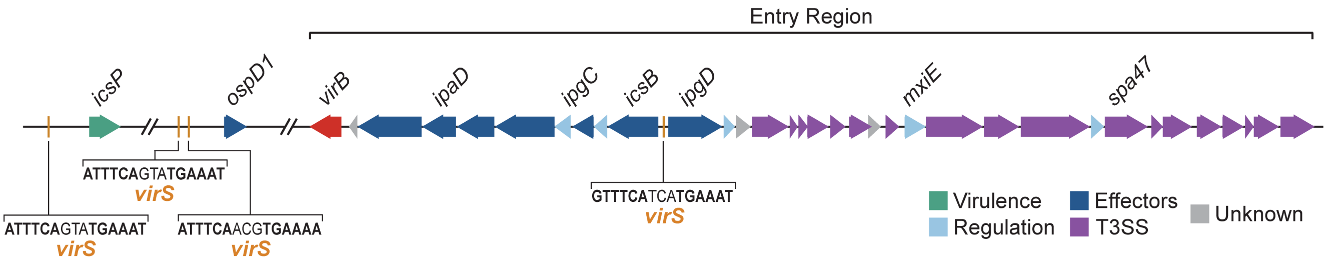

Supplementary Figure 4. Schematic of VirB-regulated genes on the pINV virulence plasmid. The biological roles of the gene products are indicated in color, as specified in the legend. The positions and sequences of the *virS* sites controlling the expression of *icsP*, *ospD1* and the two large operons in the Entry Region are indicated.

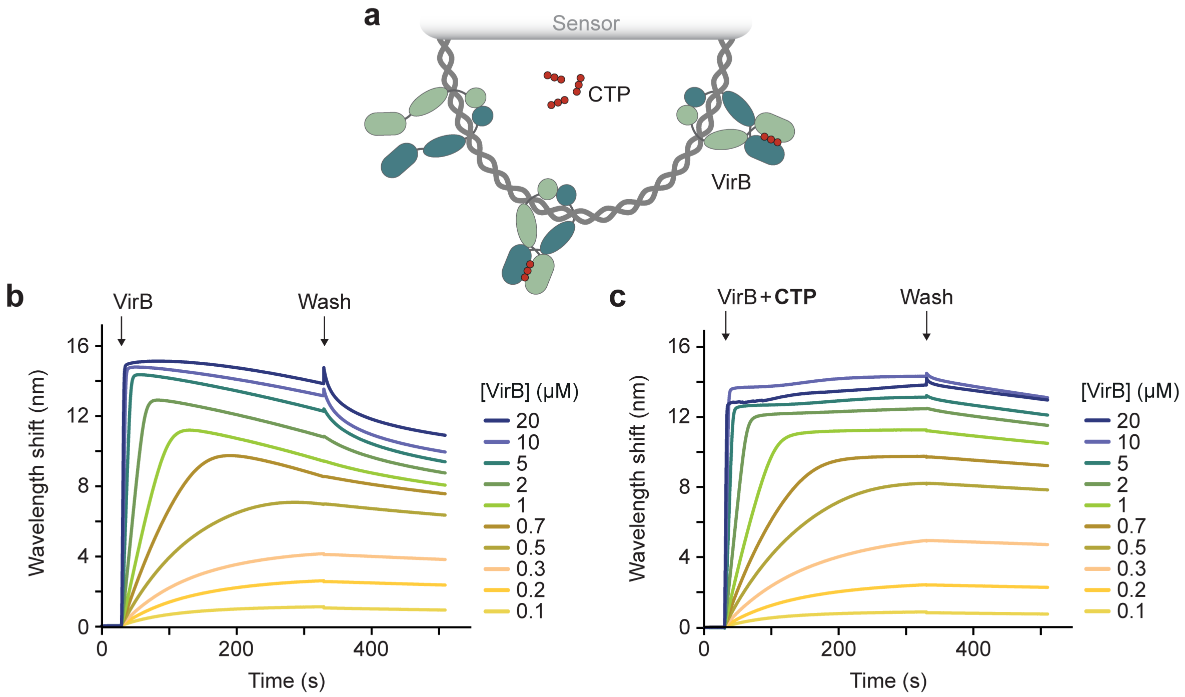

Supplementary Figure 5. Biolayer interferometry analysis of the interaction of VirB with closed non-specific DNA in low-stringency conditions. (a) Schematic of the biolayer interferometry setup used for the analyses in panels b and c. A double-biotinylated dsDNA fragment (215 bp) containing a scrambled *virS* sequence was immobilized on a streptavidin-coated biosensor and probed with VirB (green). **(b,c)** BLI analysis of the DNA-binding behavior of VirB in (b) the absence and (c) the presence of CTP (1 mM) in low-stringency buffer (150 mM NaCl). Sensors carrying the closed DNA fragment (at a density corresponding to a wavelength shift of ~1.3 nm) were probed with the indicated concentrations of VirB. At the end of the association reactions, the biosensors were transferred into protein- and nucleotide-free buffer to monitor the dissociation reactions (wash). The graphs show the results of a representative experiment (n=2-3 independent replicates).

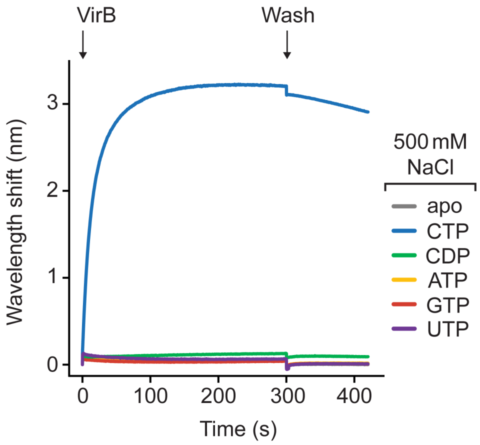

Supplementary Figure 6. Biolayer interferometry analysis of the interaction of VirB with closed, *virS*-containing DNA in the presence of different nucleotides. A streptavidin-coated biosensor carrying a double-biotinylated *virS*-containing DNA fragment (215 bp) was probed with VirB (20 µM) in the presence of the indicated nucleotides (1 mM) in high-stringency buffer (500 mM NaCl).

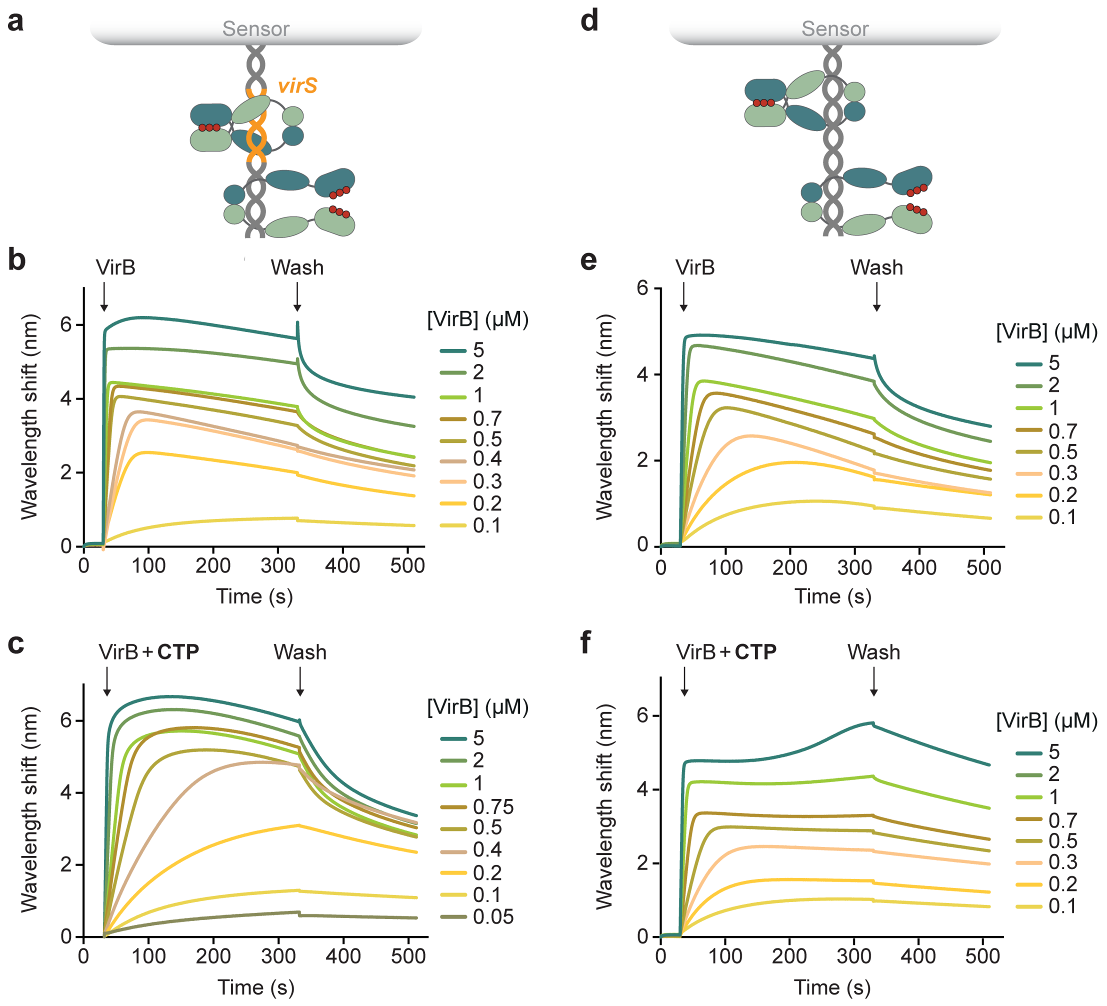

Supplementary Figure 7. Biolayer interferometry analysis of the interaction of VirB with an open double-stranded oligonucleotide in low-stringency conditions. (a) Schematic of the biolayer interferometry setup used for the analysis in panels b and c. A double-stranded *virS*-containing DNA oligonucleotide biotinylated at one of its ends was immobilized on a streptavidin-coated biosensor. **(b,c)** DNA-binding behavior of VirB in the (b) absence and (c) presence of CTP (1 mM) in low-stringency buffer (150 mM NaCl). Biosensors carrying the open *virS*-containing target DNA (at a density corresponding to a wavelength shift of ~1.5 nm) were probed with the indicated concentrations of VirB. The graphs show the results of a representative experiment (n=2-3 independent replicates). **(d)** Schematic of the biolayer interferometry (BLI) setup used for the analysis in panels e and f. A double-stranded DNA oligonucleotide containing a scrambled *virS* sequence and carrying a biotin moiety at one of its ends was immobilized on a streptavidin-coated biosensor. **(e,f)** The open, non-specific target DNA was probed with VirB in the (e) absence and (f) presence of CTP (1 mM) as described for panels b and c.

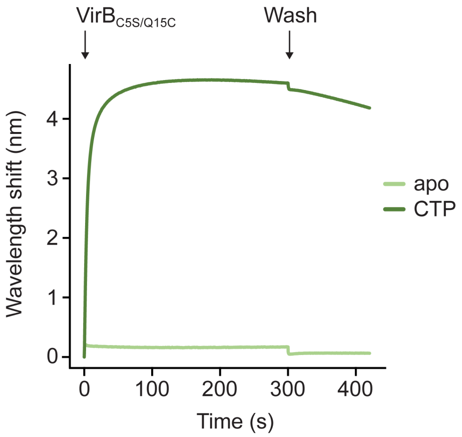

Supplementary Figure 8. Biolayer interferometry analysis of the interaction of VirB$_{C5S/Q15C}$ with closed, *virS*-containing DNA in the presence and absence of CTP. A streptavidin-coated biosensor carrying a double-biotinylated *virS*-containing DNA fragment (215 bp) was probed with VirB$_{C5S/Q15C}$ (20 µM) in the presence or absence of CTP (1 mM) in high-stringency buffer (500 mM NaCl).

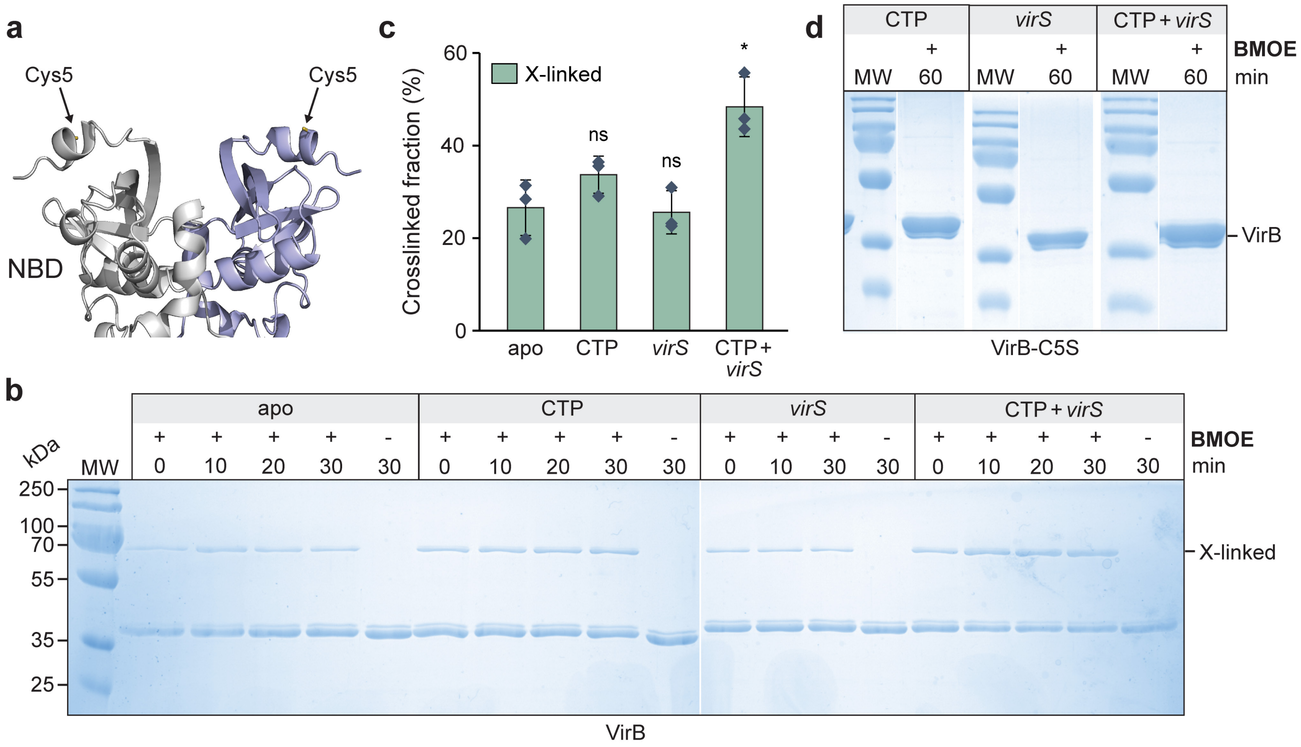

Supplementary Figure 9. *In vitro* crosslinking analysis of wild-type VirB. (a) Close-up view of the N-terminal region of the VirB dimer, as modeled by AlphaFold-Multimer (Evans et al, 2022). Arrows indicate the predicted positions of the native cysteine residues (C5). **(b)** SDS-polyacrylamide gels showing the protein species obtained in the *in vitro* crosslinking analysis. Wild-type VirB (10 µM) was incubated for the indicated time periods with CTP (1 mM), with a double-stranded DNA oligonucleotide containing a *virS* sequence (1 µM; virS-icsB-for/virS-icsB-rev) or with both CTP and *virS* DNA prior to crosslinking with BMOE and analysis of the reaction products by SDS-PAGE. Monomeric VirB and the dimeric crosslinking product (X-linked) are indicated. MW: Molecular weight marker. **(c)** Quantification of the fractions of crosslinked protein obtained in the indicated conditions (t=30 min). The columns show the mean (± SD) of three independent measurements (diamonds). *p<0.05, ns: not significant (unpaired two-sided Welch's *t*-test; versus the apo state). **(d)** SDS-gel showing the results of control *in vitro* crosslinking analyses performed with the cysteine-free VirB-C5S variant. The reactions were performed as described in panel b, with an incubation time of 60 min prior to crosslinking and SDS-PAGE analysis. Source data are provided as a Source Data file.

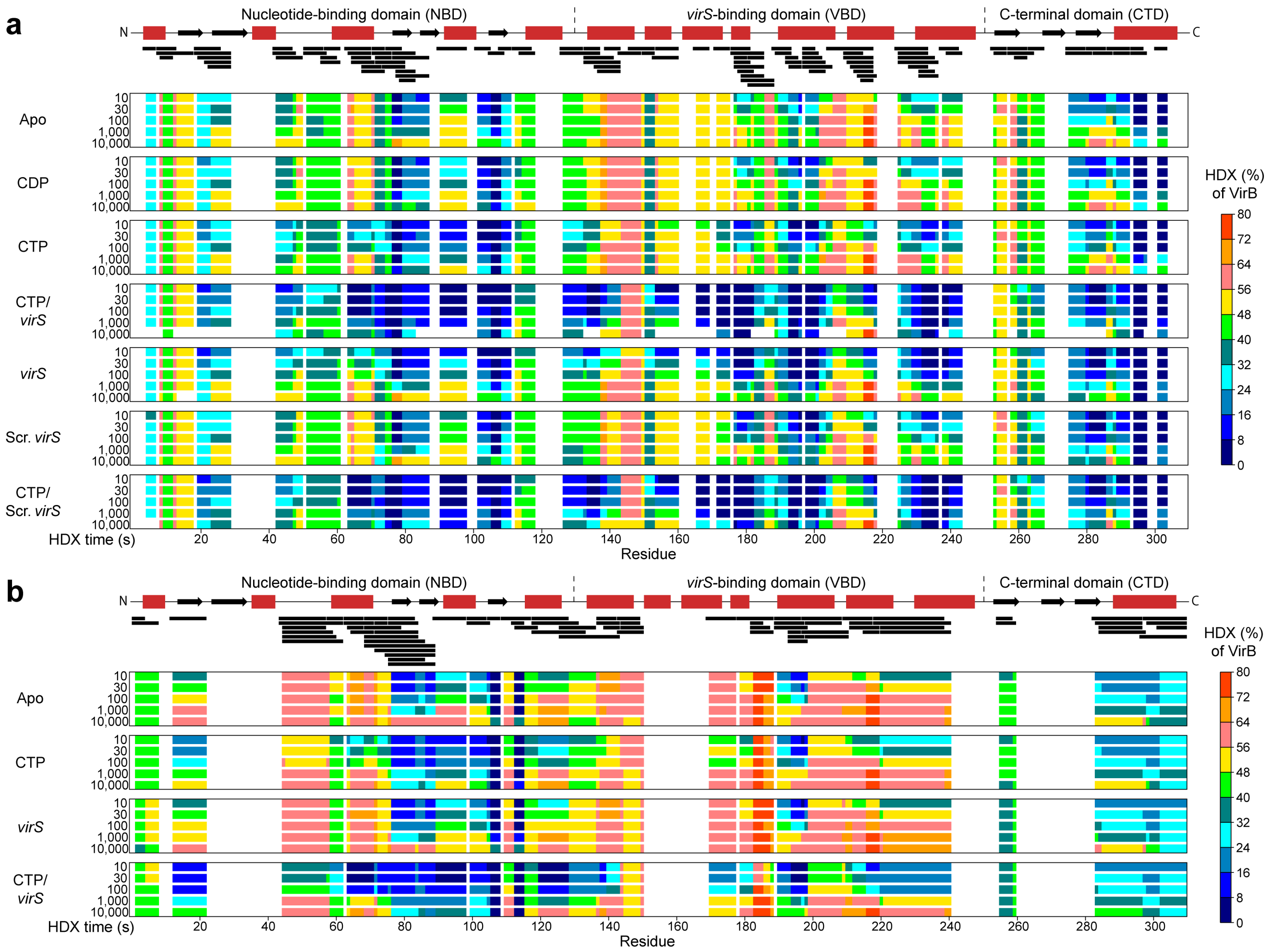

Supplementary Figure 10. Hydrogen-deuterium exchange (HDX) analysis of VirB. (a) HDX analysis of VirB in low-stringency conditions (150 mM NaCl). VirB (25 µM) was incubated in deuterated buffer for the indicated time intervals alone (apo), with double-stranded DNA oligonucleotides containing a scrambled (scrambled-virS-for/scrambled-virS-rev) or intact (virS-icsB-for/virS-icsB-rev) *virS* motif (25 µM) and/or with the indicated nucleotides (10 mM) prior to HDX analysis. Shown is the degree of HDX along the primary sequence of VirB in the indicated conditions. The color scale is given on the right. The schematic at the top displays the predicted secondary structure of VirB. The black bars represent peptides of VirB that could be analyzed for HDX. Residue-specific HDX information was obtained from these overlapping peptides by employing the shortest peptide covering any residue. No HDX could be obtained for amino acid sequences in the gaps, which indicate regions not covered by any peptides. **(b)** HDX analysis of VirB in high-stringency conditions (500 mM NaCl). VirB (50 µM) was incubated in deuterated buffer for the indicated time intervals alone (apo), with double-stranded DNA oligonucleotides containing a scrambled or intact *virS* motif (50 µM) and/or CTP (10 mM) prior to HDX analysis. The data are presented as described for panel a. Detailed information about the peptides analyzed to generate the graphs in panels a and b is given in **Supplementary Data 1**.

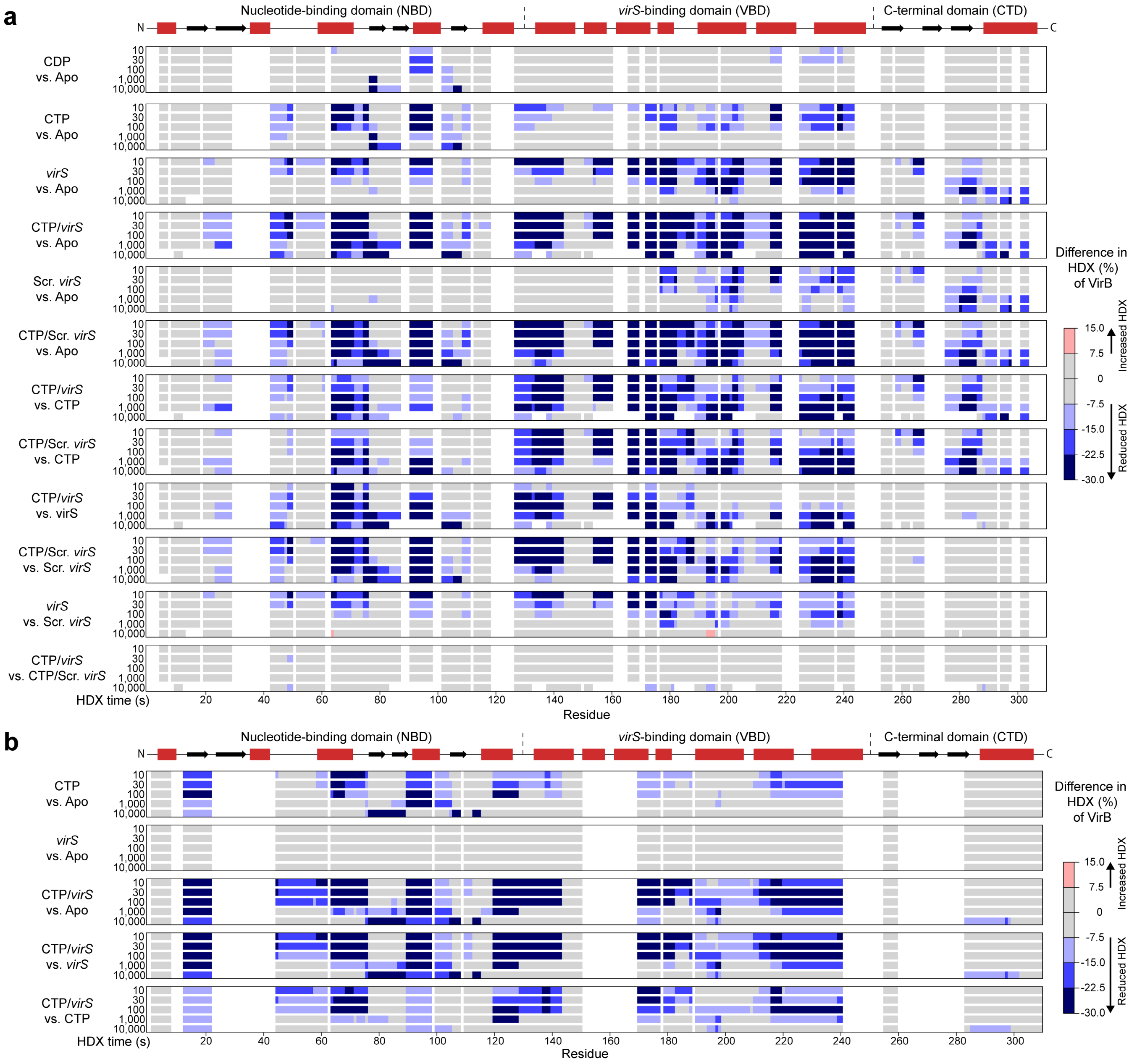

Supplementary Figure 11. Ligand-induced changes in the HDX pattern of VirB. (A) HDX analysis of VirB in low-stringency conditions (150 mM NaCl). Shown are residue-specific differences in HDX between the indicated states of VirB, projected onto the amino acid sequence of VirB. The results are derived from pairwise comparisons of the HDX data presented in **Figure S10**. The color code is given on the right. Blue color denotes reduced HDX in the first state compared to the second state in "first state" vs. "second state" comparisons. The schematic at the top shows the predicted secondary structure of VirB. **(B)** HDX analysis of VirB in high-stringency conditions (500 mM NaCl). The data are presented as described for panel a.

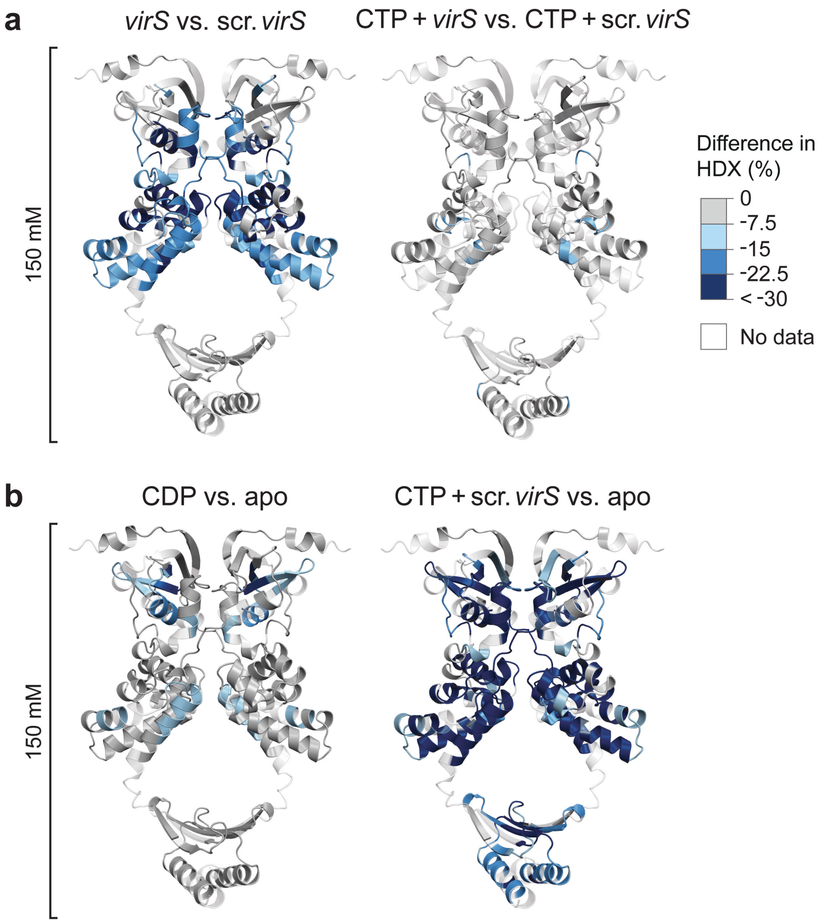

Supplementary Figure 12. Changes in the HDX pattern of VirB plotted onto the structural model of the VirB dimer. (a) Comparison of the HDX patterns of VirB with *virS*-containing DNA compared to scrambled *virS* DNA in the absence and presence of CTP in low-stringency buffer (150 mM NaCl). The color code is given on the right. Blue color denotes reduced HDX in reactions containing *virS* DNA. **(b)** Changes in the HDX pattern of VirB induced by the incubation with CDP or with both CTP and scrambled *virS* DNA compared to the apo state in low-stringency buffer. The color code is given in panel a. Blue color indicates reduced HDX in the ligand-bound state. In both panels, protein regions not covered by any peptides are displayed in transparent white.

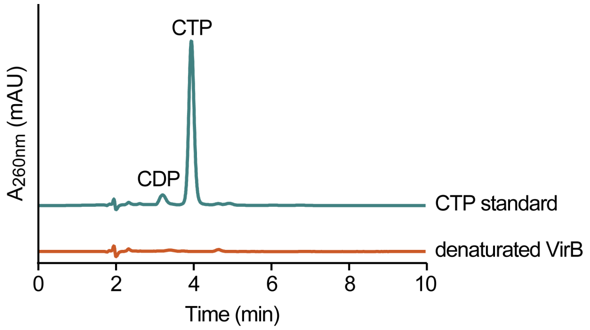

Supplementary Figure 13. Nucleotide content of purified VirB. Nucleotide content of VirB. Purified VirB (50 µM) was denatured by the addition of chloroform and heated at 95 °C to release the bound nucleotides. The aqueous phase was then analyzed for the presence of CTP or CDP by HPLC at a wavelength of 260 nm. A CTP standard (100 µM) was analyzed as a reference.

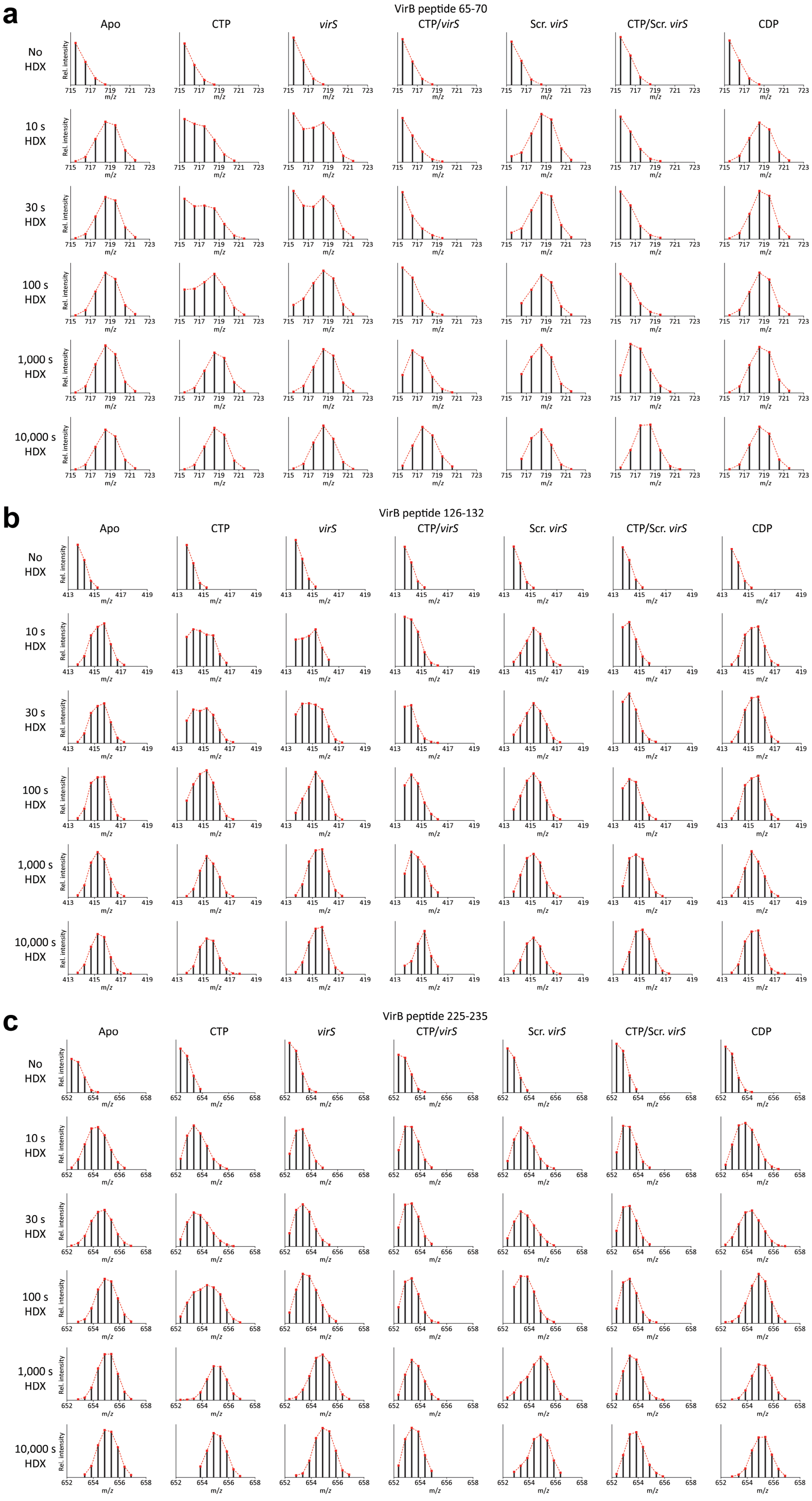

Supplementary Figure 14. Bimodal HDX behavior of VirB in low-stringency conditions. (a-c) Shown are the mass spectra (displayed as peptide ion sticks) of representative VirB peptides obtained after incubation of VirB for the specified time periods in the absence (apo) or presence of the indicated ligands in low-stringency conditions (buffer containing 150 mM NaCl). The distribution of masses indicates EX1 or mixed EX1/EX2 HDX kinetics in some of the conditions. Details of the mass spectra are given in **Supplementary Data 1**.

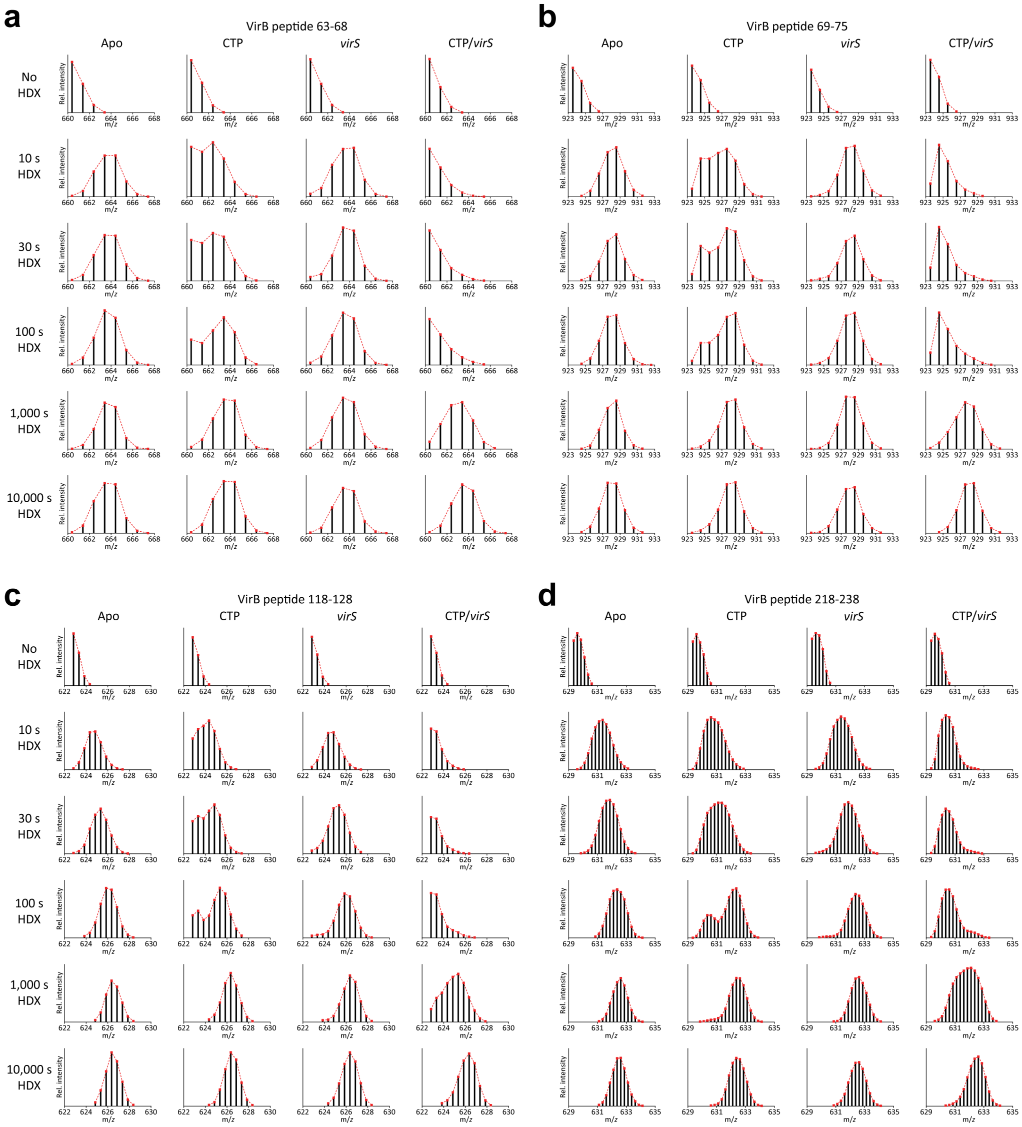

Supplementary Figure 15. Bimodal HDX behavior of VirB in high-stringency conditions. (a-d) Shown are the mass spectra (displayed as peptide ion sticks) of representative VirB peptides obtained after incubation of VirB for the specified time periods in the absence (apo) or presence of the indicated ligands in high-stringency conditions (buffer containing 500 mM NaCl). The distribution of masses indicates EX1 or mixed EX1/EX2 HDX kinetics in some of the conditions. Details of the mass spectra are given in **Supplementary Data 1**.

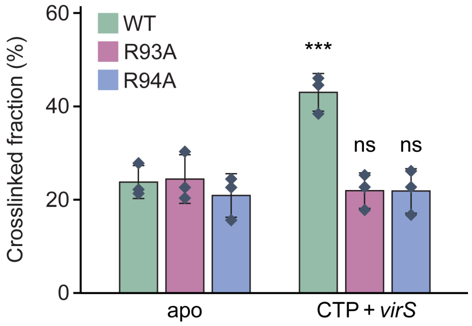

Supplementary Figure 16. ***In vitro* crosslinking analysis of wild-type VirB, $VirB_{R93A}$ and $VirB_{R94A}$.** Wild-type VirB or its mutant derivatives (10 µM) were incubated for 60 min either alone (apo) or with CTP (1 mM) and a double-stranded DNA oligonucleotide containing a *virS* sequence (1 µM; virS-icsB-for/virS-icsB-rev) prior to crosslinking with BMOE and analysis of the reaction products by SDS-PAGE. The graph shows a quantification of the fractions of crosslinked protein obtained in the indicated conditions. The columns display the mean (± SD) of three independent measurements (diamonds). *** $p<0.005$, ns: not significant (unpaired two-sided Welch's t-test; versus the apo state). Source data are provided as a Source Data file.

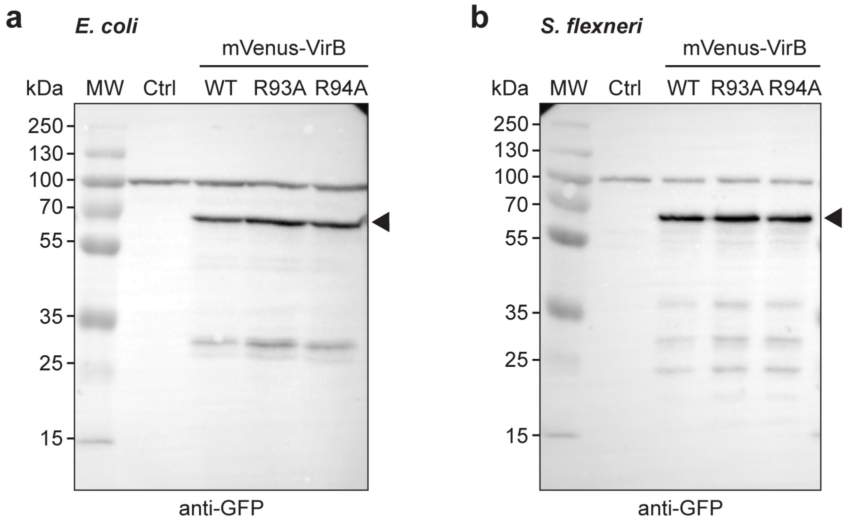

Supplementary Figure 17. Immunoblot analysis of *Escherichia coli* and *Shigella flexneri* strains producing different mVenus-VirB variants. (a) *E. coli* strains transformed with plasmids that contain the indicated *mVenus-virB* fusions under the control of an arabinose-inducible promotor (pSJ18, pSJ21, pSJ22) were grown at 28 °C for 3 h in LB medium containing 0.1% (w/v) arabinose. An uninduced culture grown in LB medium without arabinose was used as a control (Ctrl). The fusion proteins were detected with an anti-GFP antibody, which also recognizes mVenus. **(b)** *S. flexneri* strains transformed with plasmids that contain the indicated *mVenus-virB* fusions under the control of an arabinose-inducible promotor (pSJ37, pSJ38, pSJ39) were grown at 37 °C (secretion-inducing conditions) for 2 h in LB medium containing 0.2% (w/v) arabinose. A strain producing wild-type VirB under the control of an arabinose-inducible promotor (pSJ27) was used as a control (Ctrl). The fusion proteins were detected as described in panel a.

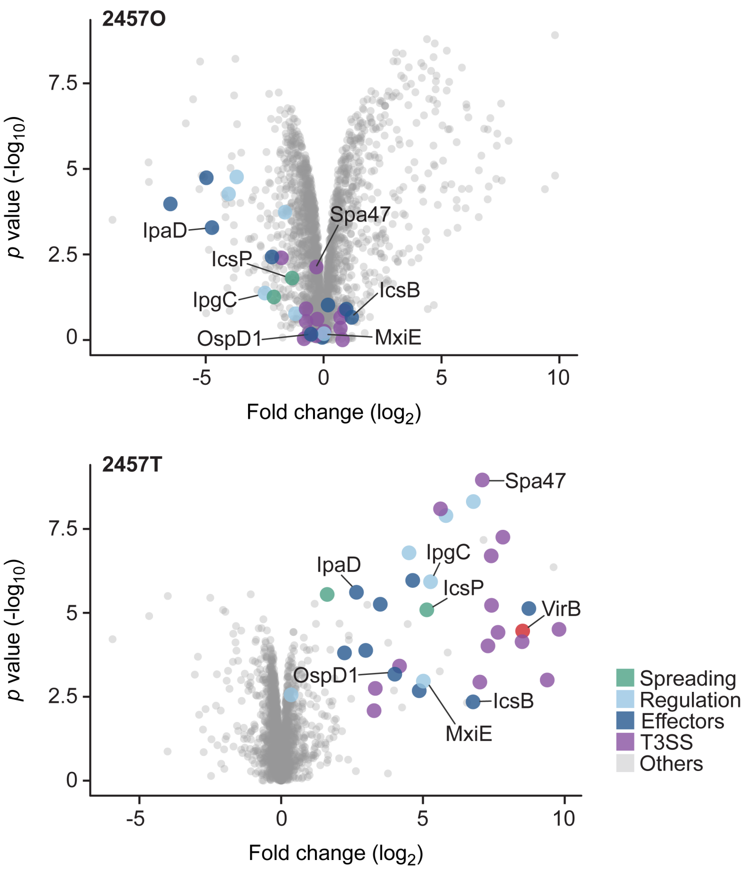

Supplementary Figure 18. Proteome analyses in *S. flexneri*. Total proteome analysis showing the accumulation of virulence proteins in different *S. flexneri* strains. *S. flexneri* 2457O, 2457T and 2457TΔ*virB* were shifted to 37 °C, cultivated in the presence of arabinose and Congo Red and subjected to total proteome analysis. Each data point in the Volcano plot represents a different protein. The x-coordinate indicates the $\log_2$ of the average difference in the peptide counts for a given protein between the indicated strain and 2457TΔ*virB*. The y-coordinate gives the $-\log_{10}$ of the corresponding p value (unpaired two-sided t-test). Known virulence factors are highlighted in color. Representative proteins are labeled. Data represent the average of three independent biological replicates. A detailed list of the data obtained in the proteomics analyses is provided in **Supplementary Data 2**.

Supplementary tables

Supplementary Table 1. Plasmids used in this study.

Plasmid	Description 1)	Construction/reference
**Plasmids used for cloning purposes**		
pBAD24-CB	P_{BAD} promoter, Amp^R	[2]
pCLF4	Plasmid carrying a Kan^R cassette flanked by FRT recombination sites	[3]
pCP20	Plasmid for the removal of resistance cassettes by FLP/FRT-mediated site-specific recombination, Amp^R, Cam^R	[4]
pKD46	Plasmid for targeted genome modifications in *E. coli* by λ red-based recombineering, Amp^R	[5]
pPR9TT	Low-copy *lacZ* reporter plasmid, Amp^R; Cam^R	[6]
pTB146	Plasmid for the overproduction of His_6-SUMO-tagged proteins under the control of the phage T7 promoter , Amp^R	[7]
**Plasmids constructed in this study**		
pSJ01	pTB146 carrying *his-sumo-virB*, Amp^R	a) PCR amplification of *virB* with primers virB-for/-rev<br>b) Digestion of pTB146 with BamHI/SapI<br>c) Insertion of the *virB* fragment into digested pTB146 by Gibson assembly
pSJ02	pTB146 carrying *his-sumo-virB*$_{C5S}$, Amp^R	Site directed mutagenesis of pSJ01 with primers virBC5S-for/-rev
pSJ05	pTB146 carrying *his-sumo-virB*$_{C5S/Q15C}$, Amp^R	a) PCR amplification of *virB-C5S/Q15C* with primers virB-for/-rev<br>b) Digestion of pTB146 with BamHI/SapI<br>c) Insertion of the *virB-C5S/Q15C* fragment into digested pTB146 by Gibson assembly
pSJ13	pTB146 carrying *his-sumo-virB*$_{R93A}$, Amp^R	a) PCR amplification of *virB-R93A* with primers virB-for/-rev<br>b) Digestion of pTB146 with BamHI/SapI<br>c) Insertion of the *virB-R93A* fragment into digested pTB146 by Gibson assembly
pSJ14	pTB146 carrying *his-sumo-virB*$_{R94A}$, Amp^R	a) PCR amplification of *virB-R94A* with primers virB-for/-rev<br>b) Digestion of pTB146 with BamHI/SapI<br>c) Insertion of the *virB-R94A* fragment into digested pTB146 by Gibson assembly
pSJ18	pBAD24-CB carrying *mVenus-virB*, Amp^R	a) PCR amplification of *mVenus* with primers pSJ18-1-for and pSJ18-3-rev<br>b) PCR amplification of *virB* with primers pSJ18-2-for and pSJ18-4-rev<br>c) Insertion of the *mVenus* and *virB* fragments into pBAD24-CB cut with NdeI and HindIII by Gibson assembly
pSJ21	pBAD24-CB carrying *mVenus-virB*$_{R93A}$, Amp^R	Site-directed mutagenesis of pSJ18 with primers virB-R93A-for/-rev
pSJ22	pBAD24-CB carrying *mVenus-virB*$_{R94A}$, Amp^R	Site-directed mutagenesis of pSJ18 with primers virB-R94A-for/-rev
pSJ27	pBAD24-CB carrying *virB*, Amp^R	a) PCR amplification of *virB* with primers pBAD-virB-for and pSJ18-4-rev<br>b) Insertion of the *virB* fragment into pBAD24-CB cut with NdeI and HindIII by Gibson assembly
pSJ28	pBAD24-CB carrying *virB*$_{R93A}$, Amp^R	Site-directed mutagenesis of pSJ27 with primers virB-R93A-for/-rev
pSJ29	pBAD24-CB carrying *virB*$_{R94A}$, Amp^R	Site-directed mutagenesis of pSJ27 with primers virB-R94A-for/-rev

1) Amp^R: ampicillin resistance, Cam^R: chloramphenicol resistance

Supplementary Table 1. Plasmids used in this study (continued).

Plasmid	Description [1)]	Construction/reference
pSJ30	pPR9TT derivative lacking the *bla* gene and carrying the *icsB* upstream region (187 bp) and the first five codons of *icsB*, Cam^R	a) PCR amplification of P*icsB* from pCP301 with primers pPR9TT-icsB-for/-rev<br>b) Insertion of the P*icsB* fragment into pPR9TT cut with BglII and HindIII by Gibson assembly<br>c) Digestion of the resulting plasmid with BsaI to remove the *bla* gene<br>d) Blunting of the ends by treatment with Klenow polymerase (NEB) in the presence of all four nucleotides<br>e) Religation
pSJ31	pPR9TT derivative lacking the *bla* gene, Cam^R	a) Digestion with BsaI to remove the *bla* gene<br>d) Blunting of the ends by treatment with Klenow polymerase (NEB) in the presence of all four nucleotides<br>c) Religation
pSJ37	pBAD24-CB carrying *mVenus-SGGGG-virB*, Amp^R	a) PCR amplification of *mVenus* with primers pSJ18-1-for and mVenus-SGGGG-virB-rev<br>b) PCR amplification of *virB* with primers mVenus-SGGGG-virB-for and pSJ18-4-rev<br>c) Fusion of the two PCR products with NdeI/HindIII-treated pBAD24-CB by Gibson assembly
pSJ38	pBAD24-CB carrying *mVenus-SGGGG-*$virB_{R93A}$, Amp^R	Site-directed mutagenesis of pSJ27 with primers virB-R93A-for/-rev
pSJ39	pBAD24-CB carrying *mVenus-SGGGG-*$virB_{R94A}$, Amp^R	Site-directed mutagenesis of pSJ27 with primers virB-R94A-for/-rev

[1)] Amp^R: ampicillin resistance, Cam^R: chloramphenicol resistance

Supplementary Table 2. Strains used in this study.

Strain	Genotype	Reference/Source
Escherichia coli		
Escherichia coli Rosetta2(DE3)pLysS	F^- *ompT* $hsdS_B(r_B^-\ m_B^-)$ *gal dcm* (DE3) pRARE2 (Cam^R)	Novagen
Escherichia coli TOP10	F^- *mcrA* Δ(*mrr-hsdRMS-mcrBC*) ϕ80*lacZ*ΔM15 Δ*lacX74 nupG recA1 araD139* Δ(*ara-leu*)7697 *galE15 galK16 rpsL*(Str^R) *endA1* λ^-	Invitrogen
***Shigella flexneri* serovar 2a**		
2457O	Avirulent derivative of strain 2457T carrying a mutant pINV plasmid that lacks most of the known virulence genes	[8], obtained from DSMZ, Germany (DSM 4782)
2457T	Virulent wild-type strain	[9], gift from Leon Schulte, Institute for Lung Research, University of Marburg, Germany
2457TΔ*virB*	Δ*virB*	This study

Supplementary Table 3. Oligonucleotides and synthetic DNA fragments used in this work.

Oligonucleotide	Sequence (in 5' to 3' direction) [1)]
AD1914	TCATCAACAGTTACATACAATCTTGCTCACACTGCATTTAACTTTTGTCAGTGCAGGCTGGAGCTGCTTC
AD1915	CGGCTGAAAGTCAAGTTCAACGAAGATTTTTATTATCTGAATTGGGCAGTCATATGAATATCCTCCTTAG
Bio-icsB-for	Biotin-TEG-AATATATTCAATTAATAAATTTAGAAACTTGAGCC
Bio-icsB-rev	Biotin-TEG-TTGTACCTCGTTTTACAACAAAAAAGATAC
BIO-TEG-scrambled-virS-for	Biotin-TEG-GCTCTGATATGAACTACATCCCA
BioTEG-icsB-for	Biotin-TEG-GCTC**GTTTCA**TCA**TGAAAT**CCCA
mVenus-SGGGG-virB-for	ATGGACGAGCTGTACAAGAGTGGAGGTGGAGGTATGGTGGATTTGTGCAACGAC
mVenus-SGGGG-virB-rev	GTTGCACAAATCCACCATACCTCCACCTCCACTCTTGTACAGCTCGTCCATGCC
pBAD-virB-for	GCTAGCAGGAGGAATTCCATATGATGGTGGATTTGTGCAACGACTTGTTAAGTATAAAGGAAGG
parS-Mxan-wt	GAGGCTTGTTCCACGTGGAACGTCGGTTTTTCGGACGTTCCACGTGGAACAAGC
pET-for	CACGATGCGTCCGGCGTAGAGGATC
pET-rev	CCTTTCAGCAAAAAACCCCTCAAGACCCG
pPR9TT-icsB-for	AGAAGGCCATCCTGACGGATGGCCTTTTTGCGTAGATCTCTTTATCTTGTGGGATTTCATGATGAAACGAGCAC TAC
pPR9TT-icsB-rev	AAAACGACGGGATCCCCCGGGCTGCAGGAATTCGATATCAAGCTTCTAATTTTGAGGATCATACTTTATTAACT CTCCATTACTTGGTGATTG
pSJ18-1-for	GGCTAGCAGGAGGAATTCCATATGATGGTGAGCAAGGGCGAGGAG
pSJ18-2-for	GTCACCGGTCGGCCACCATGGTGGATTTGTGCAACGACTTGTTAAGTATAAAGGAAGG
pSJ18-3-rev	CGTTGCACAAATCCACCATGGTGGCCGACCGGTGAC
pSJ18-4-rev	TCATCCGCCAAAACAGCCAAGCTTTTATGAAGACGATAGATGGCGAGAAATTATATCCCGAATAGCTTCATC
scrambled-virS-for	GCTCTGATATGAACTACATCCCA
scrambled-virS-rev	TGGGATGTAGTTCATATCAGAGC
virB-for	TCACAGAGAACAGATTGGTGGTATGGTGGATTTGTGCAACGAC
virB-rev	CTTTGTTAGCAGCCGGATCCTTATGAAGACGATAGATGGCGAGAA
virB-C5S-for	TGGTATGGTGGATTTGAGCAACGACTTGTTAAGT
virB-C5S-rev	ACTTAACAAGTCGTTGCTCAAATCCACCATACCA
virS-icsB-for	GCTC**GTTTCA**TCA**TGAAAT**CCCA
virS-icsB-rev	TGGG**ATTTCA**TGA**TGAAAC**GAGC
virB-R93A-for	GAAATTCTGGATGGCACTGCTAGAAGAGCATCTG
virB-R93A-rev	CAGATGCTCTTCTAGCAGTGCCATCCAGAATTTC
virB-R94A-for	TCTGGATGGCACTCGTGCAAGAGCATCTGCAAT
virB-R94A-rev	ATTGCAGATGCTCTTGCACGAGTGCCATCCAGA
virS DNA fragment (215 bp)	AATATATTCAATTAATAAATTTAGAAACTTGAGCCTGTTAACATAATCAAATTTTCTTTTGCTGTACATAATATGT ACCTCGTGAGCATATGTAGTGCTC**GTTTCA**TCA**TGAAA**TCCCACAAGATAAAGTGCCTGATGTATCAGGCTCGG AGTGTTATAGAAAAAGAGAGAACCCTGTTGAATAAGTATCTTTTTTGTTGTAAAACGAGGTACAA
scrambled *virS* DNA fragment (215 bp)	AATATATTCAATTAATAAATTTAGAAACTTGAGCCTGTTAACATAATCAAATTTTCTTTTGCTGTACATAATATGT ACCTCGTGAGCATATGTAGTGCTCTGATATGAACTACATCCCACAAGATAAAGTGCCTGATGTATCAGGCTCGG AGTGTTATAGAAAAAGAGAGAACCCTGTTGAATAAGTATCTTTTTTGTTGTAAAACGAGGTACAA

[1)] boldface: *virS* inverted repeats

Supplementary References

1. Evans R., *et al.* (2022) Protein complex prediction with AlphaFold-Multimer. bioRxiv (2022). DOI: 10.1101/2021.10.04.463034
2. Möll A. & Thanbichler M. FtsN-like proteins are conserved components of the cell division machine ry in proteobacteria. *Mol. Microbiol.* **72**, 1037-1053 (2009).
3. Santiviago C. A, Reynolds M. M., Porwollik S., Choi S.-H., Long F, Andrews-Polymenis H. L. & McClelland M. Analysis of pools of targeted *Salmonella* deletion mutants identifies novel genes affecting fitness during competitive infection in mice. *PLoS Pathog.* **5**, e1000477 (2009)
4. Cherepanov P. P & Wackernagel W. Gene disruption in *Escherichia coli*: Tc^R and Km^R cassettes with the option of Flp-catalyzed excision of the antibiotic-resistance determinant. *Gene* **158**, 9-14.
5. Datsenko K. A. & Wanner B. L. One-step inactivation of chromosomal genes in *Escherichia coli* K-12 using PCR products. *Proc. Natl. Acad. Sci. USA* 97, 6640-6645 (2000).
6. Santos P. M., Di Bartolo I., Blatny J. M., Zennaro E. & Valla S. New broad-host-range promoter probe vectors based on the plasmid RK2 replicon. *FEMS Microbiol. Lett.* **195**, 91-96 (2001).
7. Bendezu F. O., Hale C. A., Bernhardt T. G. & de Boer, P. A. J. RodZ (YfgA) is required for proper assembly of the MreB actin cytoskeleton and cell shape in *E. coli*. *EMBO J.* **28**, 193-204 (2009).
8. Mills J. A., Venkatesan M. M., Baron L. S. & Buysse J. M. Spontaneous insertion of an IS1-like element into the *virF* gene is responsible for avirulence in opaque colonial variants of *Shigella flexneri* 2a. *Infect Immun.* **60**, 175-182 (1992).
9. Formal S. B., Dammin G. J., Labrec E. H. & Schneider H. Experimental *Shigella* infections: characteristics of a fatal infection produced in guinea pigs. *J. Bacteriol.* **75**, 604-610 (1958).
