# Supplementary material for: The virulence regulator VirB from Shigella flexneri uses a CTP-dependent switch mechanism to activate gene expression
Source: Nat Commun. 2024 Jan 5;15:318. doi: 10.1038/s41467-023-44509-z (PMC10770331; doi:10.1038/s41467-023-44509-z)
Supplement: Supplementary file 3 — Description of Additional Supplementary Files [file 41467_2023_44509_MOESM3_ESM.pdf]

Description of Additional Supplementary Files:

Supplementary Data 1: Detailed description of HDX data. The spreadsheets give a summary of the conditions used for the HDX analyses and a full list of the peptides obtained in the different experiments.

Supplementary Data 2: Detailed description of shot-gun proteomics data. The spreadsheet gives a summary and a primary analysis of the shot-gun proteomics data obtained in this study.

Supplementary Data 3: Mass spectrometric identification of proteins obtained in the S. flexneri protein secretion assay. The spreadsheet indicates the gel pieces analysed and the relative abundance of the proteins identified in each of the samples.
